# Low Temperature Influence on the Behavior of Viscoelastic Layer of the Pounding Tuned Mass Damper

**DOI:** 10.3390/ma12233986

**Published:** 2019-12-01

**Authors:** Peng Zhang, Jinwei Jiang, Guangtao Lu

**Affiliations:** 1 Institute of Road and Bridge Engineering, Dalian Maritime University, Dalian 116023, China; peng.zhang47@dlmu.edu.cn; 2 Department of Mechanical Engineering, University of Houston, Houston, TX 77204, USA; 3 Key Laboratory for Metallurgical Equipment and Control of Ministry of Education, Wuhan University of Science and Technology, Wuhan 430081, China

**Keywords:** pounding tuned mass damper (PTMD), vibration control, viscoelastic material, impact fatigue, pounding, low temperature

## Abstract

In previous studies, the pounding tuned mass damper (PTMD) has been successfully demonstrated to mitigate the undesired vibration of a variety of structures at room temperature. The advantages of the PTMD over the traditional tuned mass damper (TMD) has been verified through theoretical analysis and experimental investigations. However, the PTMD relies on an impact layer made of viscoelastic material to improve its vibration control performance and robustness against detuning effect. The energy dissipation of the viscoelastic material can be affected by the changes of environmental temperature. Therefore, this paper aims to study the impact damping behavior of the viscoelastic material in the low temperature environment of the sea bed where the PTMD is expected to control vibrations of subsea pipelines. The experimental apparatus fabricated in the previous study to generate and measure the lateral impact was housed inside a refrigerator. The experimental results indicate that the pounding stiffness decreased whereas the energy dissipation increased in the low temperature environment. Moreover, an impact fatigue test was also performed in the low temperature environment and compared with the room temperature case. Experimental results from a previous study show that the viscoelastic material was damaged after 36,000 cycles of impacts in the room temperature and a cyclic hardening–softening process was observed. However, in the low temperature environment, the viscoelastic material was damaged after 50,000 cycles of impacts and the cyclic hardening–softening process was not observed. As the impact cycle grew, the pounding stiffness decreased from 53,000 N/m^1.5^ to 17,000 N/m^1.5^ and the energy dissipation increased from 46.12 J/m per cycle to 65.4 J/m per cycle.

## 1. Introduction

Undesirable vibrations occur in structures, such as buildings [1], bridges [2], engines [3,4] and aerospace structures [5,6,7], and the mitigation of these vibrations has been investigated for decades. Various vibration control techniques have been developed to reduce these unwanted oscillations [8]. In general, the vibration control techniques can be divided into four groups: active control [9,10,11], semi-active control [12,13], passive control [14,15,16] and hybrid control [17]. The tuned mass damper (TMD), which belongs to the passive control techniques, has been widely accepted for a variety of structures to mitigate wind-induced vibrations [18], seismic responses [19], vortex-induced vibrations [20] and human-induced vibrations [21], among other random excitations [22], due to its advantages of conceptual simplicity, easy installation and good effectiveness.

The earliest TMD, invented by Frahm [23], was composed of a mass block mounted on a main structure to be controlled with a specially designed spring. This device was effective in reducing the vibration near its resonance frequency. To increase the damping ability and broaden the effective band of the TMD, subsequent researchers introduced different damping components to the TMD system, such as a viscous damper [24], a friction damper [24,25,26] and eddy current elements [27,28]. Another drawback of the classical TMD is that its vibration control effectiveness will downgrade if the frequency of the TMD shifts away from the target frequency, which is termed a de-tuning effect. Consequently, a variety of active and semi-active dampers were introduced to enhance the robustness of the TMD [29,30,31,32]. However, combining with these smart dampers is financially expensive and demands continuous energy input.

**Figure 1:**
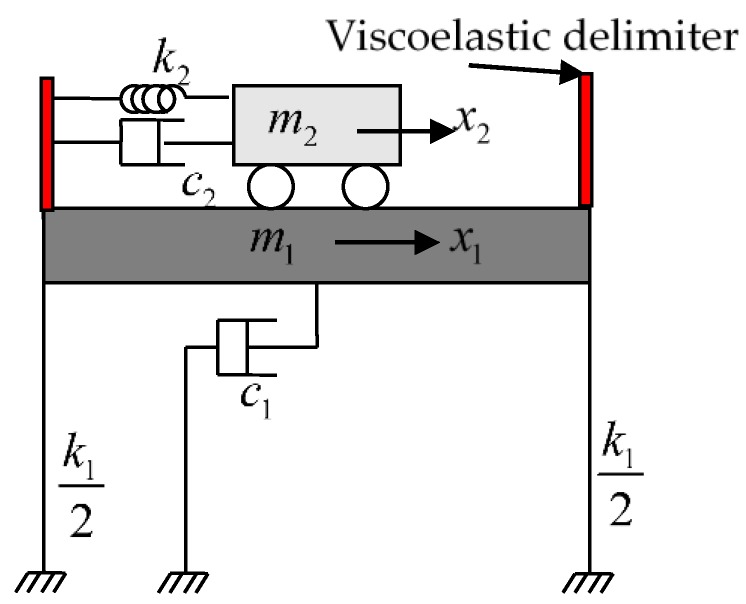
Configuration of the pounding tuned mass damper (PTMD).

In recent studies, the pounding tuned mass damper (PTMD) was proposed by introducing impact damping into the TMD system. The configuration and schematic of the PTMD is illustrated in Figure 1. When the motion of the main mass (m_1_) is slight, the motion of the tuned mass (m_2_) is also small. In this case, the tuned mass behaves like a classical TMD, which suppress the vibration of the main mass by giving it a restoring force against its motion direction. However, if the motion of the main mass exceeds a certain level, the tuned mass will impact on the delimiter and kinetic energy will be dissipated during this collision process [33,34]. The PTMD has been applied for mitigating the undesired vibration of a variety of structures, including long span power transmission tower–line systems [33,35], subsea pipeline structures [36,37,38,39], offshore platforms [40,41], high rise buildings [42,43,44,45], long span bridges [46,47], suspended building piping systems [19], and traffic signal poles [48,49]. In these literatures, the vibration performance of the PTMD and its superiority over the traditional TMD have been verified by theoretical analysis, numerical simulation and experimental studies.

As shown in Figure 1, the viscoelastic delimiter relies on a delimiter to dissipate energy via the impact between the tuned mass and the viscoelastic material. However, the impact damping behavior of the viscoelastic delimiter can be influenced by a variation of the temperature. This thermal dependent damping behavior of the viscoelastic material has been widely studied in many previous researches [50,51,52,53,54,55,56,57,58,59,60,61,62]. Several mathematical models have been proposed to consider the thermal effects and to more precisely predict the structural responses with additional viscoelastic dampers. In early studies, Chang et al. obtained empirical equations to describe the damper stiffness and loss factor [50]. Tsai [51] developed a finite-element formulation for the viscoelastic damper subjected to arbitrary loads or temperatures. Ramrakhyani et al. [54] proposed a continuously yielding element to capture the material behavior with fewer parameter. Xu et al. [55] developed a fractional-derivative equivalent standard solid model to consider influence of load frequency, amplitude and ambient temperature. Lewandowski [60] divided the models for viscoelastic dampers into two groups (i.e., the classical and nonclassical models), and compared the pros and cons of those models.

Despite the fact that extensive investigations have been carried out to reveal the thermomechanical property of the viscoelastic damper, a low temperature test is still necessary for the viscoelastic delimiter of the PTMD. In the aforementioned studies, the viscoelastic material is fabricated into sandwich type dampers, which dissipate energy via the shear deformation of the viscoelastic material. In the PTMD, however, the energy is dissipated by the compression deformation induced by impacts. This impact damping property is not yet explored in a low temperature environment. Furthermore, many intended applications of PTMD are for pipeline structures located in deep sea environment, where the temperature is around 2 °C. Therefore, it is still necessary to conduct an impact test of the viscoelastic material of the PTMD in a low temperature environment, to study the thermal influence and to extend the applications of the PTMD.

Another issue with the viscoelastic material used in PTMD is that it may undergo cycles of impacts during its long service life. An impact fatigue test has been performed to investigate the damping behavior of the viscoelastic material subjected to repeated poundings [63]. However, this study was conducted in room temperature. Thus, it is still necessary to carry out an impact fatigue test in a low temperature environment.

This paper aims to study the impact damping property of the viscoelastic material in a PTMD in the low temperature environment. The paper is organized as follows: After this introduction, a nonlinear pounding force model is revisited, preparing parameters to characterize the impact damping capacity of the viscoelastic material. Subsequently, a description of the experimental device and the test procedure is presented in Section 3. Further, experimental results of the low temperature case are compared with the room temperature case to reveal the influence of thermal effect. Conclusive findings and suggestions for future work are provided as a closure.

## 2. Impact Damping Property of the Viscoelastic Material in a PTMD

Since the energy dissipation pattern of the PTMD is different from the sandwich type viscoelastic damper, parameters such as storage modulus and loss modulus are not suitable to characterize the damping property of the viscoelastic material in the PTMD. Pounding stiffness and the energy consumed during each pounding are defined to interpret the damping behavior of the viscoelastic material.

### 2.1. Pounding Stiffness

The pounding stiffness is a parameter defined in a previous study [33] to predict the nonlinear pounding force. In this pounding force model, the pounding stiffness can also indicate the energy consumption ability. The mathematical expression is as follows:

(1) $$F=\{\begin{array}{c} \beta \delta^{3/2}+c\dot{\delta }\;(\dot{\delta }>0)\\ \beta \delta^{3/2}\;\left(\dot{\delta }\leq 0\right) \end{array}$$

in which δ and $\dot{\delta }$ are the impact depth and its velocity; β is the pounding stiffness which can be attained using the displacement and the pounding force recorded in the impact test, with the Curve Fitting Toolbox embedded in MATLAB/Simulink; and c is the pounding damping which can be computed by:

(2) $$c=2\xi \sqrt{\beta \delta \frac{m_{1}m_{2}}{m_{1}+m_{2}}}$$

where $m_{1}$ and $m_{2}$ are the masses of the two colliding bodies, and ξ is the impact damping ratio.

### 2.2. Energy Dissipated per Impact Cycle

In order to compare the impact damping ability of the viscoelastic material in a room temperature and low temperature environment, the energy dissipated during each pounding can be attained as follows:

(3) ΔW=∫Fδdδ

where ΔW is the energy dissipated during each pounding; F denotes the nonlinear pounding force; and δ is the impact depth. Since ΔW can be influenced by the impact intensity, a normalized energy dissipation $\Delta \bar{W}$ is defined as follows:

(4) $$\Delta \bar{W}=\Delta W/\delta_{\max}$$

where $\delta_{\max}$ is the maximum value of the impact depth.

## 3. Experimental Setup

**Figure 2:**
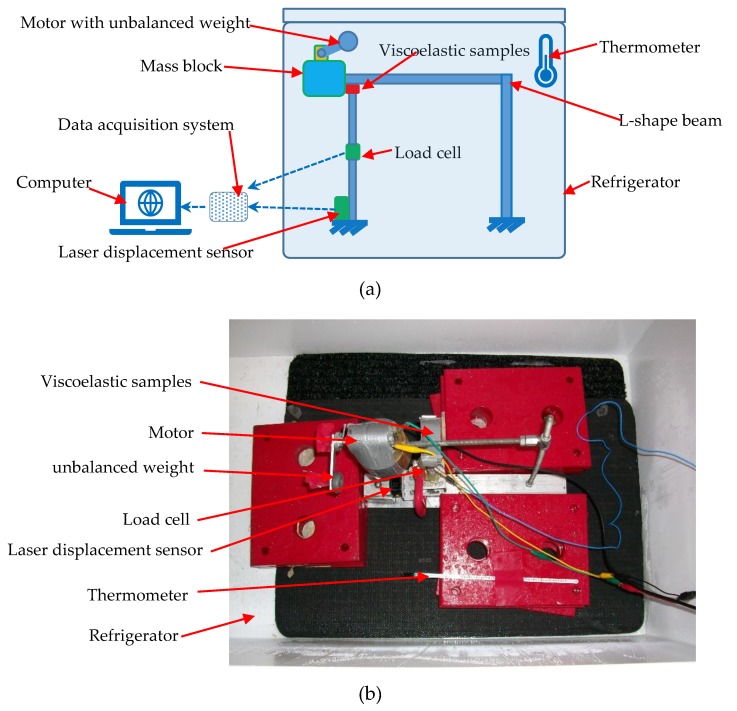
Experimental setup: (**a**) schematic; (**b**) experimental device inside the refrigerator.

Figure 2 illustrates the experimental setup. The impact tester shown in Figure 2a had a configuration similar to that of the PTMD designed for pipelines structures. One major part of the tester was an L-shape beam with a mass block (1.5 kg) fixed at the cantilever end. The mass vibrates vertically and pounds on the viscoelastic delimiter beneath it, when the motor (Type# 1105, produced by Pololu) rotates an unbalanced mass. A force sensor and a noncontact displacement sensor were employed to record the pounding force and the relative displacement. The sampling frequency was set to 1 kHz. The experimental apparatus was assembled inside a refrigerator. A thermometer was also put in the refrigerator to makes sure that the testing temperature was around 2 °C.

## 4. Results and Discussion

### 4.1. Impact Damping Behavior in Low Temperature

**Figure 3:**
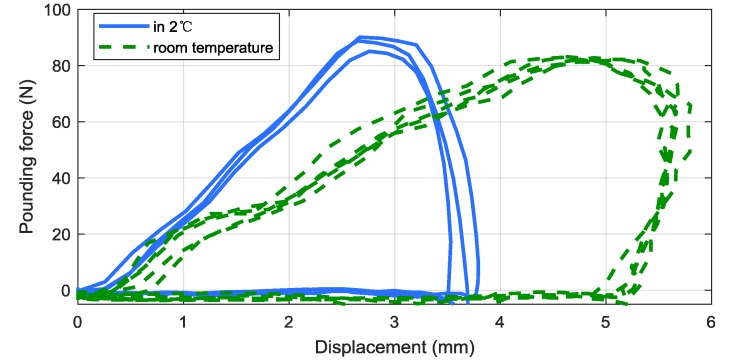
Hysteresis loops in room temperature and low temperature environments.

An impact test was performed with the viscoelastic material, using the experimental device illustrated in Figure 2. Due to the limited space inside the refrigerator, only the PTMD damper was installed. The hysteresis loops of the specimen in room temperature and in low temperature are compared in Figure 3. As illustrated in the figure, the maximum displacement was around 3.5 mm in the low temperature environment, which is smaller than that of the room temperature (5 mm) under similar pounding force level. This indicates that the pounding stiffness will be increased in a low temperature environment. Moreover, the slope of solid line (low temperature) is steeper than the dashed line (room temperature), also implying that the pounding stiffness will be increased in a low temperature environment. It can be observed that the area surrounded by the hysteresis loops of the low temperature case is smaller than that of the room temperature case, indicating that impact damping will be decreased in a low temperature environment. It should be noted that the frequency response function (FRF) is not included in this study, due to the limitation of the testing device. The FRF of the PTMD damping system shall be investigated to further demonstrate the dynamic property, with upgraded experimental apparatus.

The impact stiffness and the energy dissipated during each pounding were also used to compare the pounding behavior of the samples in room temperature and low temperature environments. The estimated pounding stiffness in the 2 °C environment was 53,225 N/m^1.5^, while the pounding stiffness in the room temperature was 17,259 N/m^1.5^ [33]. This indicates that the pounding stiffness will be increased when the temperature is decreased. In previous studies [63], the $\Delta \bar{W}$ of the room temperature condition was attained to be 51.29 J/m, whereas $\Delta \bar{W}$ of the low temperature case was decreased to 46.12 J/m. This also implies that the energy dissipated via impacts of viscoelastic material will decrease in low temperature environments.

### 4.2. Impact Fatigue Test in Low Temperature

In practical engineering, PTMDs are often used for vibration control of structures constructed in remote and harsh environments (e.g., offshore platforms and power transmission tower in mountain areas). It will be very difficult, if possible, to maintain a PTMD designed for these structures. Consequently, the PTMD is expected to function well for a long service life. Therefore, this study performed an impact fatigue test in a low temperature environment to investigate impact fatigue behavior of the viscoelastic material. The impact fatigue test was conducted as shown in Figure 2. As illustrated in the figure, when the motor rotates the unbalanced weight, the L-shape beam will vibrate vertically and continuously impacts on the viscoelastic samples beneath it, causing impact fatigue of the viscoelastic material. A similar experimental study was conducted in room temperature. In this study, the impact fatigue test was conducted in the low temperature, and the experimental results are presented and compared with the room temperature case.

#### 4.2.1. Appearance

**Figure 4:**
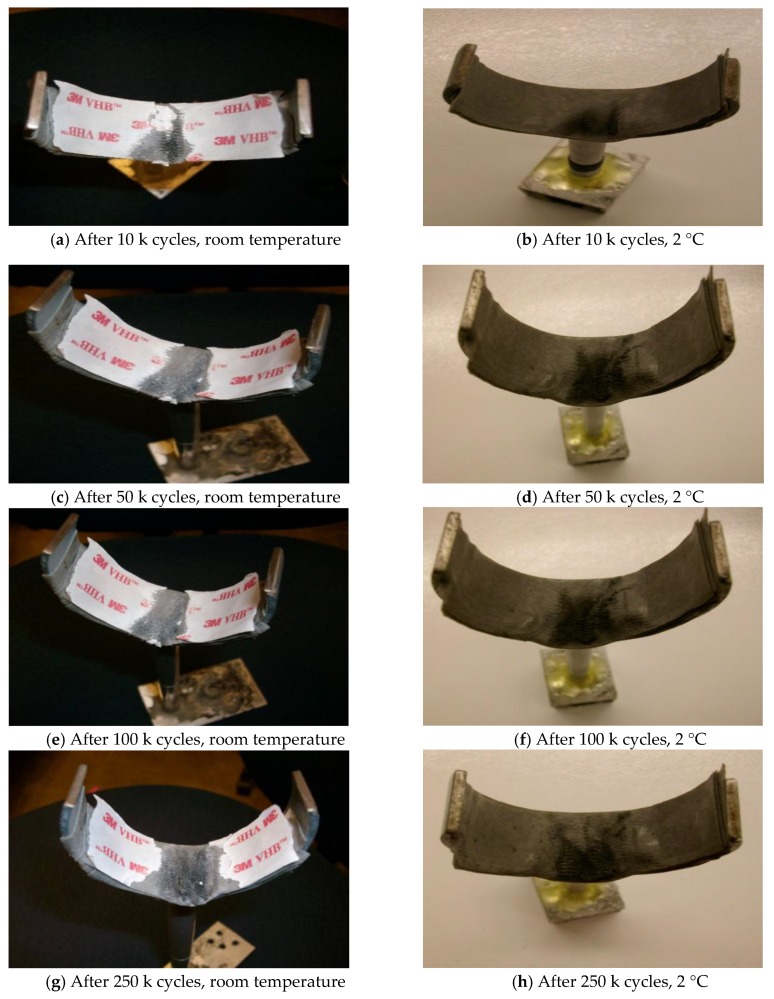

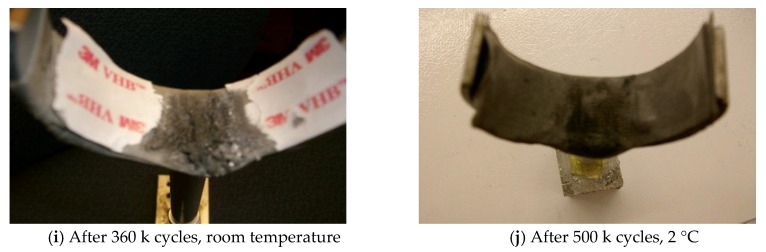
Appearance of the sample after cyclic impacts.

During the low temperature impact fatigue test, the appearance of the viscoelastic material was photographed after every 10,000 impacts. The pictures are compared with the room temperature case in Figure 4. One common phenomenon, which was observed in both the room temperature case and the low temperature case, was that the thickness of the viscoelastic sample was decreased after repeated poundings and the damage of the viscoelastic material is more visible as the number of the impacts increases. The difference between the two cases is that the damage of the low temperature case was less severe than the room temperature case under same cycles of impacts. As shown in Figure 3i, which is the room temperature case, the viscoelastic material was severely damaged and the metal was exposed in some areas after only 360,000 cycles of impacts. In the low temperature case (Figure 3j), the viscoelastic material was damaged to a similar level after 500,000 cycles of impacts.

#### 4.2.2. Pounding Stiffness

**Figure 5:**
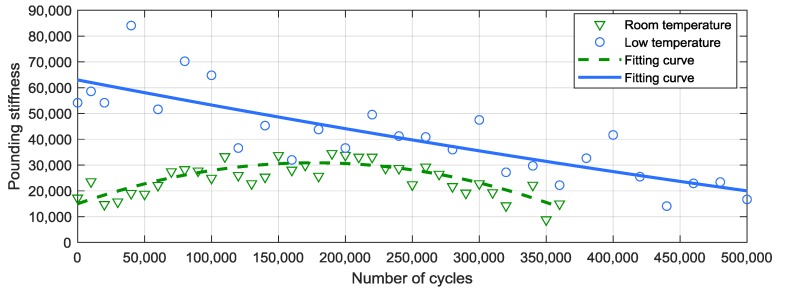
Pounding stiffness after cyclic impacts.

In Figure 5, the pounding stiffness, *β*, after continuous poundings in room temperature and in low temperature environment is illustrated. A cyclic-hardening and cyclic-softening process can be observed in the room temperature case. As shown by the triangle and the green dash line, *β* was increased from 15,000 N/m^1.5^ to 35,000 N/m^1.5^ after the first 180,000 strokes; after that, the pounding stiffness was decreased to 9000 N/m^1.5^ [63]. However, in the low temperature environment, *β* decreases from 53,000 N/m^1.5^ to 17,000 N/m^1.5^ by the continued impacts; no cyclic-hardening and cyclic-softening process can be observed.

#### 4.2.3. Damping Capacity

**Figure 6:**
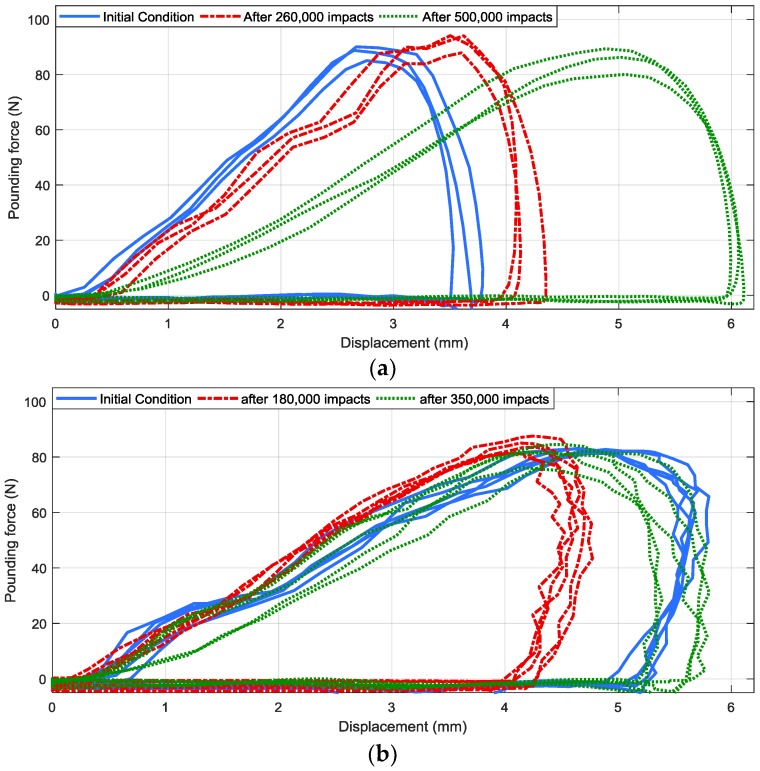
Hysteresis loops after repeated poundings: (**a**) in 2 °C, (**b**) in room temperature.

In order to further investigate the damping capacity of the specimen subjected to continued impacts in low temperature environment, the hysteresis loops are compared in Figure 6. Figure 6a corresponds to the low temperature case. The hysteresis loops of the room temperature case are also provided in Figure 6b for comparison. As shown in Figure 6a, the maximum impact depth (the largest deformation of the viscoelastic material) grew from around 3.5 mm to around 6 mm as the impact cycles increased from 0 to 500,000. In Figure 6b, which is the room temperature case, the maximum impact depth was first reduced from 5.6 mm (initial condition) to 4.7 mm (after 180,000 strokes), then increased to 5.8 mm (after 350,000 strokes).

Energy dissipation ability can also be reflected by these hysteresis loops of Figure 6. The area surrounded by the red lines in Figure 6a are larger than the blue lines (before the impact fatigue test), which indicates that the energy dissipation was enlarged by the repeated impacts in low temperature environment. Whereas in the room temperature case (Figure 6b), the surrounded area firstly decreased when impact cycles grew from 0 to 180,000, then increased as impact cycles grew to 350,000.

**Figure 7:**
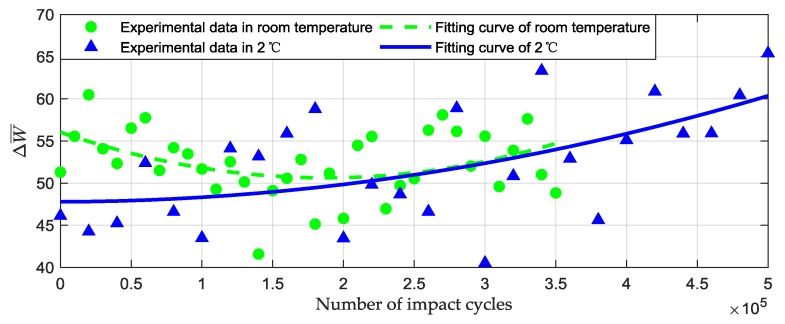
Dissipated energy during each pounding in different temperature.

Figure 7 illustrates the normalized energy dissipation ability after cyclic poundings. The green rounds and the green dashed line are the experimental results and a fitting curve of the room temperature environment. The dissipated energy per impact cycle $\Delta \bar{W}$ decreased from 51.29 J/m per cycle (initial condition) to 45.14 J/m per cycle (after 180,000 impacts), and then increased to 48.83 J/m per cycle (after 35,000 impacts). However, this decrease and increase process was not observed in the low temperature environment. As shown by the experiment results and fitting curve of 2 °C (the blue triangle and the solid blue line), $\Delta \bar{W}$ increased from 46.12 J/m per cycle to 65.4 J/m per cycle after 500,000 cycles of impacts.

## 5. Conclusions and Future Work

In this study the impact damping behavior of the viscoelastic material of the PTMD in a low temperature environment (2 °C, deep sea floor temperature) was investigated. A specially designed experimental device was established in a refrigerator. An impact test and an impact fatigue test were conducted with this tester. The pounding stiffness, hysteresis loops and the energy consumed during each pounding were applied for interpreting the pounding damping property of the viscoelastic material used in the PTMD. Conclusions drawn from experimental results are summarized as follows:

1. When ambient temperature drops from room temperature to 2 °C, the pounding stiffness of the viscoelastic material is significantly increased, while its energy dissipation ability is slightly decreased, indicating that the PTMD can be applied for vibration control of structures in the low temperature environment.
2. In low temperatures, continuous pounding will cause impact fatigue of the viscoelastic material of the PTMD, with its pounding stiffness gradually reduced and the energy dissipation ability increased. This is different from the room temperature case.

Due to the limitation of the experimental apparatus, impact tests were only conducted at 2 °C. In future studies, the ambient temperature shall be set to other levels to further explore the relation between temperature and impact damping behavior. Empirical formulas and analytical solutions shall be developed to assist design of PTMD for other temperatures. A primary structure to be controlled with a PTMD shall also be installed inside a larger refrigerator to perform forced vibration tests under varied excitation frequencies and amplitudes. The frequency response function can further demonstrate the dynamic property of the PTMD damping system.
